# Effects of invisible lip movements on phonetic perception

**DOI:** 10.1038/s41598-023-33791-y

**Published:** 2023-04-20

**Authors:** W. Teramoto, M. O. Ernst

**Affiliations:** 1 Faculty of Humanities and Cultural Sciences (Psychology), Kumamoto University, 2-40-1 Kurokami, Kumamoto, 860-8555 Japan; 2 Applied Cognitive Psychology, Ulm University, Albert-Einstein-Allee 43, 89081 Ulm, Germany

**Keywords:** Consciousness, Perception, Human behaviour

## Abstract

We investigated whether ‘invisible’ visual information, i.e., visual information that is not consciously perceived, could affect auditory speech perception. Repeated exposure to McGurk stimuli (auditory /ba/ with visual [ga]) temporarily changes the perception of the auditory /ba/ into a ‘da’ or ‘ga’. This altered auditory percept persists even after the presentation of the McGurk stimuli when the auditory stimulus is presented alone (McGurk aftereffect). We used this and presented the auditory /ba/ either with or without (No Face) a masked face articulating a visual [ba] (Congruent Invisible) or a visual [ga] (Incongruent Invisible). Thus, we measured the extent to which the invisible faces could undo or prolong the McGurk aftereffects. In a further control condition, the incongruent faces remained unmasked and thus visible, resulting in four conditions in total. Visibility was defined by the participants’ subjective dichotomous reports (‘visible’ or ‘invisible’). The results showed that the Congruent Invisible condition reduced the McGurk aftereffects compared with the other conditions, while the Incongruent Invisible condition showed no difference with the No Face condition. These results suggest that ‘invisible’ visual information that is not consciously perceived can affect phonetic perception, but only when visual information is congruent with auditory information.

## Introduction

Speech perception is important in human social life. While the auditory modality provides the primary information to speech perception, the visual modality also contributes considerably. An excellent example is the McGurk effect, in which participants report hearing a new sound (‘da’ or ‘ga’) when a spoken /ba/ syllable is dubbed onto a visual presentation of [ga]^1^. Recent research has shown that audiovisual integration can occur without conscious awareness^2–6^. Here, we defined conscious awareness or awareness (of stimuli) as a reportable subjective experience (of stimuli) (see^7^ for further discussion about how awareness/unawareness is defined or measured operationally). Several studies have investigated audiovisual integration without visual awareness for speech perception^8–10^. Using the continuous flash suppression (CFS) technique^11^, Palmer and Ramsey^9^ reported that invisible lip movements influenced visual attention guidance when congruent auditory stimuli were concurrently presented but did not influence auditory speech perception (i.e., McGurk effects). They suggested that the information of the influencing modality must be consciously perceived to affect the perception of another modality; specifically, visual information needs to be consciously perceived to affect auditory speech perception. Contrary to their suggestion, however, Plass et al.^10^ showed that invisible lip movements could facilitate auditory perception of the corresponding spoken word using a spoken word categorization task (target words or non-target words). The authors explain the difference from Palmer and Ramsey^9^ from the level-of-processing viewpoint: visual information without awareness can modulate auditory speech perception at the level of word encoding but not syllable encoding.

Roles of awareness in information integration have been one of matters of debate in scientific literatures for a long time (^12^ for a review). Mudrik et al.^12^ suggest that, mostly, conscious awareness is needed to integrate novel combination of features from different sensory modalities into a unified percept, but is not necessary to integrate those features whose association is previously learned through multiple exposure (but see Discussion for exceptional cases). If this suggestion holds, whether invisible information can affect auditory speech perception or not should be dependent on the specific combination of audiovisual features: conscious awareness is not necessary for integration of audiovisual features that observers commonly integrate (i.e., that are congruent). Specifically, in a case of the McGurk effect, congruent audiovisual features such as auditory /ba/ and visual [ba] should be integrated even when visual [ba] is rendered invisible. In contrast, incongruent audiovisual features such as auditory /ba/ and visual [ga], which typically induce the McGurk effect, should not be integrated when visual [ga] is rendered invisible, as those multisensory features are incongruent. Thus, different from Plass et al.’s^10^ suggestion, this view suggests that invisible visual signals can affect auditory speech perception even at the level of syllable encoding.

This study aimed to investigate the effects of invisible visual information on auditory speech perception at the level of syllable encoding. To do so we relied on the McGurk aftereffect^13–15^. The McGurk aftereffect is a phenomenon in which, after the exposure to McGurk stimuli (auditory /ba/ + visual [ga]), the auditory syllable that is originally perceived as /ba/ is continued to be perceived as ‘da’ or ‘ga’ (we write the perceived syllables by enclosing single quotations) for several seconds after the visual stimulus disappears, presenting only the auditory signal alone (phonetic recalibration). Thus, the perception of the auditory syllables can be changed temporarily by recalibration, but if not stabilized it will return to the original ‘ba’ perception. Particularly it will return to the original ‘ba’ percept if congruent audiovisual stimuli are presented, while it will be stabilized if intermittently incongruent audiovisual stimuli are again presented. We speculated that invisible visual information is not sufficiently strong to modulate auditory speech signals at the level of syllable encoding so that the McGurk effect is not induced as shown in Palmer and Ramsey^9^. However, if the auditory speech signals become more ambiguous and thus unstable by the McGurk aftereffect, even invisible visual information may affect the auditory speech process. Further, the repeated exposure to the McGurk stimuli for the McGurk aftereffect might make the incongruent audiovisual stimuli less incongruent, resulting in their integration in the following invisible situation.

In the experiments, after the sequential presentation of McGurk stimuli consisting of eight different speakers (induction phase), the same auditory syllables used in the induction phase were presented with or without low-contrast visual lip movements masked by dynamic noise (test phase). If unconscious visual stimuli are effective for audiovisual integration at the level of syllable encoding, the lip movements congruent with the auditory syllables (Congruent Invisible condition) should reduce the McGurk aftereffects in the test phase compared with the condition without lip movements (No Face condition). Conversely, those with the incongruent auditory syllables (Incongruent Invisible condition), which typically induces the McGurk effects in visible situations, should stabilize the illusion thus prolong the effect. We found that the invisible lip movements influenced on the perception of auditory syllables only when the audiovisual signals were congruent in Experiment 1. We did not find the difference between the Incongruent Invisible and the No Face conditions in Experiment 1; however, the incongruent lip movements might have contributed to the stabilization of the McGurk aftereffects to some extent. If the number of trials after the adaptation induction increased, the difference between the Incongruent Invisible and No Face conditions might be pronounced. Therefore, Experiment 2 further investigated the effect of incongruent lip movements on auditory speech perception by prolonging test trials (five epochs; 8 trials each) after the aftereffect induction. Nevertheless, no difference was found between the Incongruent Invisible and No Face conditions.

## Methods

### Participants

Thirty-two volunteers (17/15/0 women/men/diverse, mean age: 24.7 ± 4.3 [standard deviation] years) and 21 volunteers (12/9/0 women/men/diverse, mean age: 26.0 ± 5.1 years) were recruited as participants for Experiments 1 and 2, respectively. Eight participants of Experiment 1 also participated in Experiment 2. All participants had a normal or corrected-to-normal vision and normal hearing. Informed consent was obtained from all participants. Participants were aware of the study purpose, risks and benefits but had no knowledge about which stimuli were presented in each trial. The sample size was calculated using G*Power 3^16,17^ to achieve a power of 0.8 with an effect size of 0.25 (medium effect) in Experiment 1; this indicated 28 participants. Predicting a dropout rate of around 15%, we recruited 32 participants. As for Experiment 2, the results of Experiment 1 showed a strong effect size for the effect of condition (partial eta squared = 0.51), which required only nine participants to achieve a power of 0.8. However, we recruited approximately twice as many participants, including several dropouts, to find even a small difference in either epoch. The study design was approved by the Ethics Committee of the Faculty of Humanities and Social Sciences, Kumamoto University, and the experiments were performed under the principles of the Declaration of Helsinki.

### Apparatus and stimuli

Visual stimuli were presented on a 19-inch color LCD monitor (60 Hz refresh rate; 1280 × 1024 resolution) using a personal computer (MacBook Pro, OSX) and MATLAB R2014b (MathWorks, Natick, MA) with the Psychophysics Toolbox extensions^18,19^. Images for the left and right eyes were presented in the left and right halves of the monitor, respectively, and brought to the eyes via a mirror haploscope. The viewing distance was 57 cm. A chinrest was used to stabilize the participant’s head. Sounds were presented via headphones (SENNHEISER HD569).

**Figure 1:**
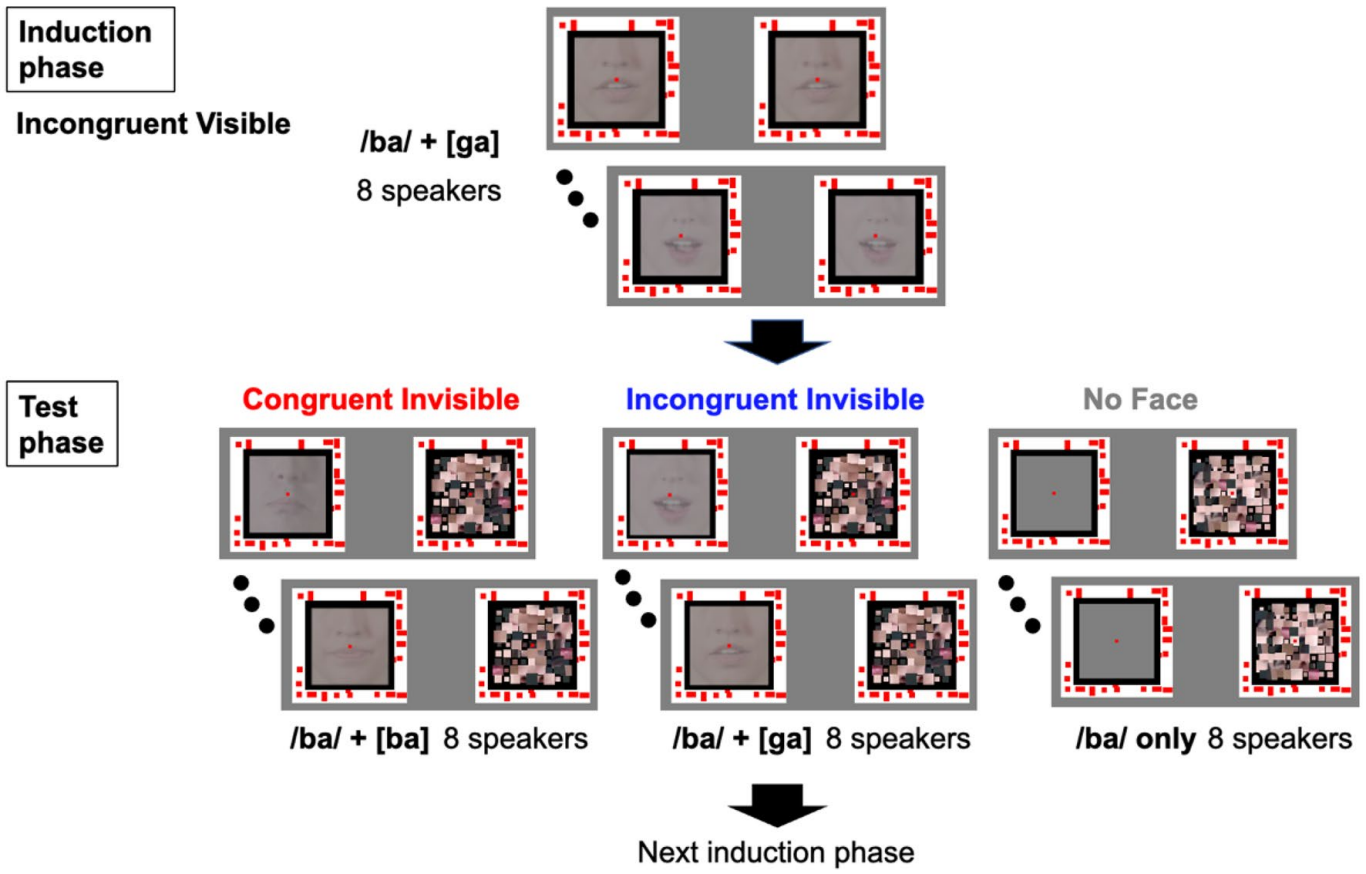
Schematic diagram of experimental stimuli and procedure. In the Induction phase, eight visible McGurk stimuli with different speakers were sequentially presented (once per speaker) to induce the McGurk effects and the aftereffects. In the Test phase, eight invisible face stimuli with different speakers (visual [ga] and visual [ba] for the Incongruent Invisible and Congruent Invisible conditions, respectively) or no stimuli (No Face condition) were sequentially presented with the dynamic mask. In both Induction and Test phase the participants were instructed to judge which syllable they had heard—'ba’, ‘da’, or ‘ga’—and whether they saw the speaker’s face during the trial. Note that the data for the Induction phase were regarded as the baseline, Incongruent Visible condition.

The audiovisual materials were obtained from the Beauchamp Lab website (https://openwetware.org/wiki/Beauchamp:McGurkStimuli)^20^. McGurk stimuli (auditory /ba/ combined with visual [ga]) and audiovisual congruent stimuli pronouncing /ba/ (auditory /ba/ combined with visual [ba]) from four male and four female speakers were used. The length of each movie was 2.6 s. Pink noise was added (signal-to-noise ratio: − 3 dB) to each movie sound (sampling frequency: 48 kHz) to enhance the McGurk effect^21^ and its aftereffects. The maximum sound pressure level of the speakers ranged from 50.7 dBA to 57.0 dBA. The movies were converted from 29.97 Hz to 60 Hz. The face of each speaker was cropped with a square mask (5.4° × 5.4°), showing the mouth region, and the center of each image was placed just above the upper lip (Fig. 1). The face had a mean luminance of 47.1 cd/m^2^ (maximum and minimum luminance of 67.4 cd/m^2^ and 11.3 cd/m^2^, respectively), and was presented against a black background (0.0 cd/m^2^; 6.3° × 6.3°). Further, the image presented to each eye was surrounded by a white frame (8.1° × 8.1° and 0.9° thick) textured by binocularly matched red square dots (19.5 cd/m^2^; 0.36° × 0.36° each; 33% of density) to facilitate stable binocular fusion. These “fusion facilitating frames” were presented throughout the experiment. The other part of the screen was colored gray (17.0 cd/m^2^). The dynamic mask for CFS was presented to the dominant eye. A dynamic mask was generated using one of the speakers’ original face images. The image was cut into 9 × 9 (0.6° × 0.6°) parts. At every 100 ms (i.e., 10 Hz), the position of each part was shuffled and randomly displaced in the range of ± 0.15°, and each size was also randomly changed (0.6° ± 0.3°). The random variables were drawn from uniform distributions.

### Procedure

There were three invisible conditions in both Experiments 1 and 2: Congruent Invisible, Incongruent Invisible (McGurk), and No Face. The auditory stimulus was /ba/ in all trials, while the visual stimulus was either [ba] for Congruent Invisible trials, [ga] for Incongruent Invisible trials, or not present for No Face trials. A further control condition, “Incongruent Visible,” in which the incongruent faces remained unmasked was also included as a baseline and for the adaptation (the details are described below). Each condition was presented as a block.

#### Experiment 1

Eight visible McGurk stimuli with different speakers were sequentially presented (once for each speaker; the order of the speakers was randomized) in the first half of the block. This had two roles: one was to measure McGurk effect as a baseline (Incongruent Visible), and the other was to induce the aftereffects (induction phase). Subsequently, in the second half of the block, eight invisible face stimuli (once for each speaker; the order of the speakers was randomized) for either invisible condition were sequentially presented with the dynamic mask (the test phase). This combination of the Incongruent Visible and either invisible condition was repeated nine times in each experimental session (i.e., three blocks for one invisible condition). Two experimental sessions were conducted. The order of the condition blocks was randomized for each participant. Thus, for each participant, the number of trials per each invisible condition was 48 (8 trials × 6 blocks), while that for the Incongruent Visible (induction phase) was 144 (8 trials × 18 blocks).

Before the experiment, we assessed each participant’ ocular dominance using the Miles test^22,23^, and set the program so that the dynamic visual noise was presented on the dominant eye. Additionally, we adjusted the angles of the mirrors of the haploscope to each participants’ eyes so that presented visual images were fused. At the beginning of each trial, a blue fixation cross (0.27° × 0.27°) was presented with the fusion facilitating frames and the characters of “B,” “D,” and “G.” These characters were used to remind the participants of what they were required to discriminate. One second later, the characters were replaced with either the identical faces for both eyes in the induction phase or with the dynamic mask for the dominant eye and the face for the non-dominant eye in the test phase. The face was first blurred but faded in over a 2000 ms period for the both phases. This was to reduce the likelihood that it would break through the dynamic mask to become visible in the test trials (the same procedure was used in the induction phase). When this fade-in period was completed, the color of the fixation square changed to red and, simultaneously, a warning tone (400 Hz, 0.1 s) was presented to inform the participants that the articulation movie would start. After the articulation movie was finished, a red fixation and another display asking for participants’ responses were presented until the participant responded using a standard numeric keypad: In all trials, including those in the induction phase, participants were instructed to mention which syllable they had heard—'ba’, ‘da’, or ‘ga’—and whether they saw the speaker’s face at any time during the trial. Note that we asked them to press the ‘Yes’ (face visible) key even at the slightest hint of the face. The responses for the induction phase were analyzed as Incongruent Visible condition. In Experiment 1 mean rates (± standard deviations) of reporting seeing faces were 0.9% (± 2.2%) and 1.0% (± 2.1%) across all participants in the congruent and incongruent conditions, respectively. As these numbers are very low the CFS worked very well. The trials participants reported seeing faces did not feed into the analysis.

#### Experiment 2

The induction phase of eight trials for the Incongruent Visible condition was followed by 40 trials of either invisible condition, in which a set of eight different speakers was repeated five times (the order of speakers was randomized in each set). Three different invisible conditions were conducted in different sessions. The order of the sessions was randomized for each participant. The total was 40 trials for each invisible conditions and 24 trials for the Incongruent Visible condition.

In the test trials of Experiment 2, after the face was faded in, participants were required to press a key to indicate that they saw the dynamic mask (i.e., no face was seen). If they saw the face instead of the dynamic mask, they were required to wait until they saw the dynamic mask. Furthermore, if they responded that they saw the face after the trial, they would need to return to the beginning of the session (i.e., induction phase). We took this procedure to prevent the “breaking the masking” trials from topping up the adaptation effect. The other procedure was the same as in Experiment 1.

#### Both experiments

Before any experimental sessions, each participant completed approximately 32 trials to familiarize the tasks, using a program in which all four conditions were randomly presented. Participants were also instructed to focus on the fixation point throughout the experimental session, although no gaze monitoring system was used. No feedback was provided to the participants throughout the experiments. To enforce participants’ attention to the center of the face (mouth region), participants were also instructed to count the number of color changes of the fixation (from red to brief green flashes for 33.3 ms), occurring in several trials in a session 250–300 ms after the face was faded in. At the end of each session, the experimenter asked the number. Some participants reported a few trials short or over, but all participants reported close to the number of color changes which actually occurred (the average absolute differences [actual number − reported number] were 1.75 ± 1.62 in Experiment 1 and 0.94 ± 1.64 in Experiment 2). Therefore, we did not exclude any participants from the following analysis using this score. Experiments 1 and 2 took approximately 45 or 30 min, respectively, to complete the sessions, including breaks.

### Statistical analyses

#### Experiment 1

As indicated above, trials in which participants reported seeing faces in the ‘invisible’ trials were removed from the analysis in Experiment 1. Participants who reported seeing faces in more than 20% of the trials were excluded from the analysis. This occurred for three out of 32 participants, leaving 29 participants for analysis. In each condition for each participant, the proportion of ‘da’ or ‘ga’ responses (i.e., illusory percepts) was calculated. A one-way repeated measures ANOVA was performed after arcsine transforming the proportion data for the invisible conditions in order to stabilize the variance.

#### Experiment 2

One participant was removed because the faces were hard to be masked. An epoch was defined as a sequence of eight different speakers. There were five epochs because a set of eight different speakers was repeated five times after the adaptation in this experiment. The proportion of ‘da’ or ‘ga’ responses in each epoch was calculated in each condition for each participant. After arcsine transformation was conducted on the proportion data, a two-way repeated measures ANOVA with two within-participant factors (condition and epoch) was performed. Even if the interaction was not emerged, we analyzed the simple effect of condition in each epoch (i.e., interaction effect) with Bonferroni correction to investigate how long the effect found in Experiment 1 lasted.

For both experiments, the Shapiro–Wilk test was used to check the normality of the residuals after the arcsine transformation. Mendoza's Multisample Sphericity Test assessed whether the assumption of sphericity was met for ANOVA. When the assumption of sphericity was violated, the degree of freedom was adjusted by using Greenhouse–Geisser’s epsilon. The Holm-Bonferroni method was used for multiple comparison. The alpha level for statistical tests was set at 0.05. The analyses were performed using the R software package^24^.

## Results

### Experiment 1

**Figure 2:**
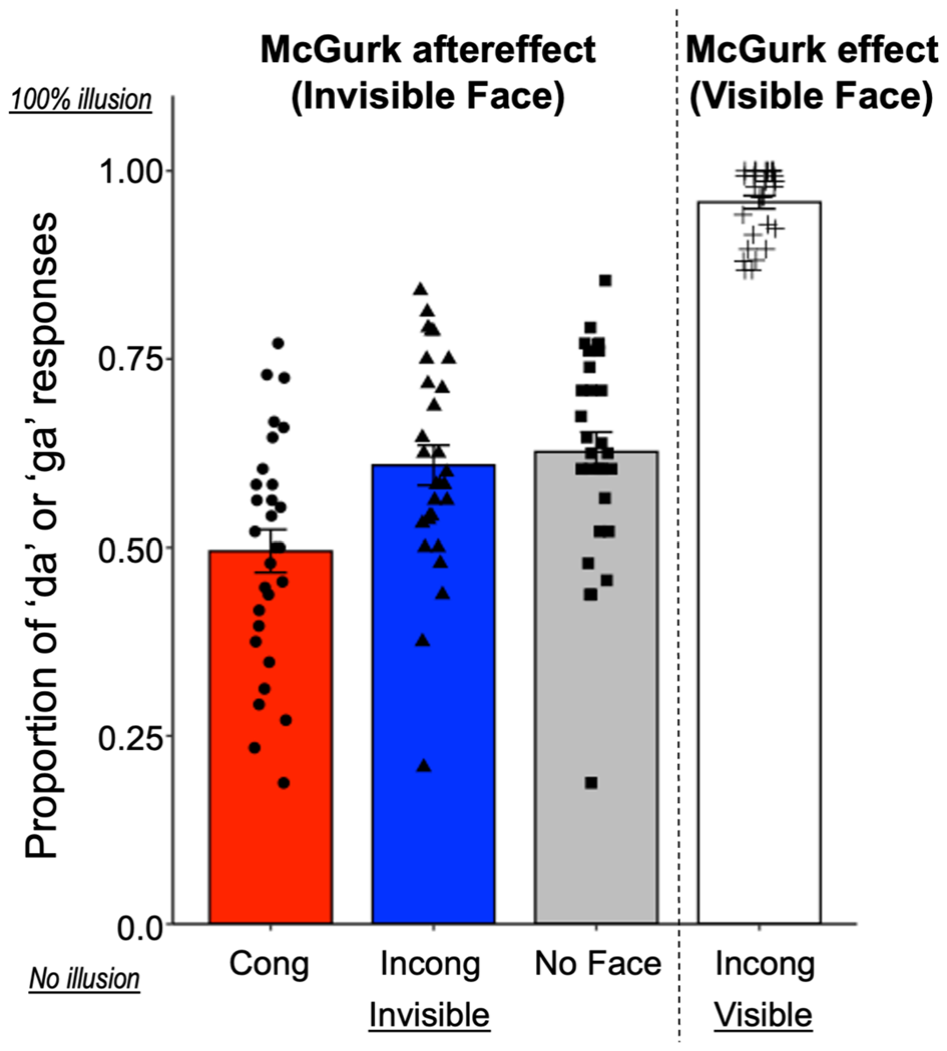
Proportion of ‘da’ or ‘ga’ response (illusory percepts) in Experiment 1. The rightmost bar (Incong Visible) represents the proportion in the McGurk induction phase. Black circles, triangles, squares, and crosses represent individual data. Error bars denotes the standard error of the mean. Plots were generated using R software version 4.0.5 (R Core Team (2021). R: A language and environment for statistical computing. R Foundation for Statistical Computing, Vienna, Austria. http://www.R-project.org/).

Figure 2 shows the proportion of reporting ‘da’ or ‘ga’ across the participants in each condition of Experiment 1 together with the responses in visible trials in the induction phase (rightmost plot). As shown in Fig. 2, the McGurk effects were almost perfectly induced in the Incongruent Visible condition (i.e., induction phase). The proportion of reporting ‘da’ or ‘ga’ was lower in the Congruent Invisible condition than the other conditions. This result was statistically confirmed. An ANOVA revealed a significant effect of condition (*F* (2, 56) = 29.20, *p* < 0.001, η^2^ = 0.140). The following multiple comparisons revealed significant differences between the Congruent Invisible and the other conditions (*p* < 0.001), but not between the Incongruent Invisible and No Face conditions (*p* = 0.264). This indicates that lip movements congruent with the auditory syllable can reduce the McGurk aftereffects even when they are suppressed from awareness. Conversely, no difference was found between the incongruent and no-face conditions, suggesting that the incongruent lip movements do not seem to have the ability to enhance the McGurk aftereffects when the participants were not aware of them.

**Figure 3:**
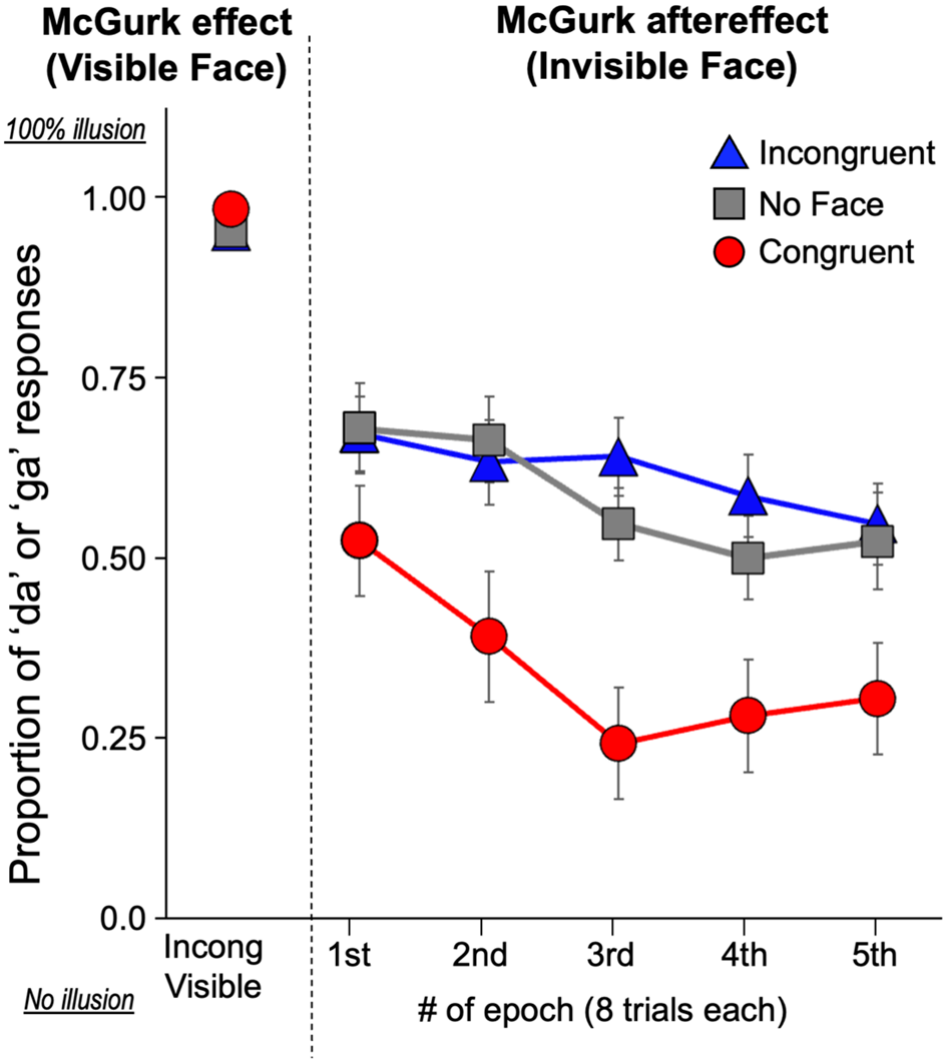
Mean proportion of ‘da’ or ‘ga’ response (illusory percepts) as a function of epoch (8 trials of different speakers). The leftmost plots are for the baseline, Incongruent Visible condition. Error bars denotes the standard error of the mean.

Figure 3 shows the results of Experiment 2. A two-way ANOVA revealed significant main effects of condition (*F* (1.52, 28.80) = 18.81, *p* < 0.001, η^2^ = 0.156) and epoch (*F* (2.40, 45.64) = 7.11, *p* = 0.001, η^2^ = 0.035), but not for the interaction effect (*F* (4.22, 80.23) = 1.51, *p* = 0.204, η^2^ = 0.015). The multiple comparison for the effect of condition revealed that the proportion of ‘da’ or ‘ga’ responses were lower for the Congruent Invisible condition than the other conditions. A (planned) post hoc test with Bonferroni correction for the interaction revealed that a significant effect of condition in all epochs except for epoch 1 (epoch 1: *F* (1.44, 26.80) = 2.69, *p* = 0.511, η^2^ = 0.068; epoch 2: *F* (1.15, 3.18) = 6.85, *p* = 0.0145, η^2^ = 0.155; epoch 3: *F* (1.49, 28.26) = 18.07, *p* < 0.001, η^2^ = 0.287; epoch 4: *F* (1.45, 27.54) = 10.14, *p* = 0.007, η^2^ = 0.191; epoch 5: *F* (1.36, 25.88) = 9.92, *p* = 0.010, η^2^ = 0.135). Multiple comparison for the data of epochs 2, 3, 4, and 5 revealed that the proportion of reporting ‘da’ or ‘ga’ was significantly lower in the Congruent Invisible condition than the other conditions, but no difference between the Incongruent Invisible and No face conditions. Thus, even with prolonged test trials after the aftereffect induction (five times more test trials than those in Experiment 1), the effect of invisible face on phonetic perception did not change; the influence of incongruent face was never pronounced even when time passed after the adaptation, if it was presented invisible. This suggests that incongruent invisible faces do not have an ability to stabilize the McGurk aftereffects.

## Discussion

This study investigated whether invisible lip movements can affect auditory speech perception. In both experiments, after the McGurk aftereffects were induced using visible lip movements articulating [ga] with the auditory /ba/ signals (i.e., auditory /ba/ signals came to be perceived as ‘da’ or ‘ga’) in the induction phase, the same auditory signals were presented in conjunction with movies of lip movements masked by CFS. Experiment 1 demonstrated that the McGurk aftereffects could be reduced when the invisible lip movements were congruent with the auditory /ba/ as compared with when they were incongruent with the auditory syllable (i.e., [ga]) or when no lip movements were presented. Conversely, no differences were observed between the incongruent and no-face conditions. Experiment 2 demonstrated that when the face was presented invisible and incongruent its influence was never pronounced even when time has passed between its presentation and the adaptation. These results suggested that lip movements can affect phonetic perception at the level of syllable encoding even when they do not reach visual conscious awareness at least for audiovisual speech stimuli that have been typically used (i.e., auditory /ba/ and visual [ga]).

Our finding contrasts suggestions by Palmer and Ramsey^9^ that the information of the influencing modality must be consciously perceived to affect the perception of another modality. However, several previous studies using visual capture effects have already shown that invisible information can alter sound perception (e.g.,^3,4^). These findings, together with those of this study and Plass et al.^10^, suggest that audiovisusal interaction can occur regardless of whether information on the influencing modality is consciously perceived. Plass et al.^10^ suggested that visual information without awareness can modulate auditory speech perception at the level of word encoding but not syllable encoding. However, our findings indicate that it can modulate auditory speech perception even at the syllable encoding level or phonetic categorization. Several studies have shown that consciously perceived stimuli can increase perception of congruent stimuli masked by CFS stimuli (e.g.,^2,25^). For example, in the audiovisual speech literature, Alsius and Munhall^2^ reported that audible stimuli can break CFS masking into congruent visual stimuli more quickly than incongruent visual stimuli. Thus, it is conceivable that the congruent audiovisual stimuli in the present study could almost have been processed at a conscious level, so that they are available for further processing, as compared with the invisible incongruent audiovisual stimuli (see the last paragraph of this section for further discussion on this issue). As we presented only one syllable, it may have been too short to reach conscious access.

Notably, our results support the hypothesis by Mudrik et al.^12^ that conscious awareness is unnecessary for integration of signals that is strongly associated by their repeated, paired presentation for a prolonged period but needed for integrating signals which have hardly been accompanied. Indeed, the strong association between auditory /ba/ + visual [ba], which has been learned through everyday lives, weakened the phonetic perception (‘ga’ or ‘da’) temporally constructed by short-term exposure to visible McGurk stimuli (auditory /ba/ + visual [ga]) (i.e., McGurk aftereffect). Conversely, the incongruent audiovisual signals (auditory /ba/ + visual [ga]) did not enhance the illusory phonetic perception (‘da’ or ‘ga’). Note that no enhancement of the McGurk aftereffects in the Incongruent condition could not be due to the ceiling effect, given that the incongruent audiovisual signals (auditory /ba/ + visual [ga]) almost perfectly induced the McGurk illusion when they were visible. An absence of the McGurk effect (i.e. not its aftereffect) under the invisible condition has been reported thus far^8,9^. Munhall et al.^8^ used a dynamic version of Rubin’s face-vase illusion^26^ in which the face appeared to articulate syllables [aga]. This was presented with auditory /aba/. They showed that the McGurk effect occurred only when participants consciously perceived the face, instead of vase, in moving face-vase stimuli. These findings indicate that conscious awareness is probably necessary to connect arbitrary auditory and visual signals, i.e., the newly formed audiovisual association.

One may argue that the incongruent audiovisual signals in the test phase were not novel to be exact, because these signals was conjointly presented several times in the McGurk aftereffect induction phase. Thus, it should have affected task performance in the invisible situation as well, if Mudrick et al.’s^12^ suggestion holds true. This might be a matter of how many times the pair is presented beforehand. Specifically, the number of trials in the induction phase might be too small to establish a strong association enough to influence the following invisible trials. Studies showed that extensive repetition of an arbitrary pair of features can form a new association, even if the participants were not aware of the association (e.g.,^27,28^). Thus, even with incongruent audiovisual signals (auditory /ba/ + visual [ga]), increasing the presentation repetition of the pair might induce more integration of these signals in the invisible situation, thus, ‘ga’ or ‘da’ percepts, than in current study.

Several studies have reported that different neural mechanisms are involved in processing incongruent (McGurk) and congruent audiovisual speech stimuli^29–31^. Erickson et al.^29^ reported that the left posterior superior temporal sulcus (pSTS), repeatedly reported as a key region for the McGurk percept^32–34^, was recruited for the congruent audiovisual speech stimuli, while the left posterior temporal gyrus (pSTG) was activated during the McGurk stimuli. Erickson et al.^29^ suggest that some brain areas, including the left pSTS, are crucial for integrating audiovisual speech signals, while other brain areas, including the left pSTG, were more important for generating the merged percept. Our results may reflect the differences in the neural mechanisms between congruent and incongruent audiovisual stimuli. Perhaps, integrating audiovisual speech signals in the pSTS can occur before conscious visual awareness.

This study has some limitations. Firstly, we used only audiovisual stimuli, which are typically used to achieve the McGurk effect. Therefore, it is unclear whether the effect observed in this study can be generalized to other audiovisual speech stimuli. Secondly, we defined awareness of stimuli by participants’ subjective, dichotomous reports (‘visible’ or ‘not visible’) so that “brief glimpses”^35^ might occur. Ramsøy and Overgaard^35^ reported that there is a level of awareness at which participants do not recognize the stimulus but are aware of “something being there” and that this level of awareness of stimuli can contribute to better-than-chance-level performance under “unaware” situations in the dichotomous reports. Thirdly, related to this issue, there is a possibility that participants were reluctant to honestly report the visibility of stimuli in Experiment 2 because they would not have proceeded further with the trials if they reported the face as visible. Thus, it is necessary to use other methods to ensure that participants are not aware of the stimuli. These issues should be further investigated in future studies.

## Conclusions

Our results suggest that lip movement information can affect phonetic perception before participants have clear experience of visual stimuli only when a strong association between the visual and auditory speech signals has already been established. This is the case at least for audiovisual speech stimuli that have been typically used (i.e., auditory /ba/ and visual [ga]). This strongly supports the hypothesis by Mudrik et al.^12^. While the neural mechanisms underlying the present effects remain open, our findings suggest that audiovisual speech signals can be integrated during relatively early visual processing.

## Data Availability

For scientific purposes the datasets generated and analyzed during this study are available from the corresponding author upon reasonable request.
